# The Association Between Elevated Body Mass Index and Clinical and Surgical Outcomes in Patients Undergoing Robot-Assisted Radical Prostatectomy: A Retrospective Study

**DOI:** 10.7759/cureus.112229

**Published:** 2026-07-07

**Authors:** Pedro Henrique Silva Carvalho, Carlos Eduardo da Silva, Gabriel Cunha Garcia Capretz, Nicholas Tavares Lemos, Maria Eduarda Figueiredo Severiano Alves, Augusto Fernandes Custodio Araujo, Giovanna Lamounier Linck, Túlio Mendes Batista, Giovana Vilas Boas Caetano, Alexandre Teruya, Eunice Harue Higuchi, Sabrina Thalita dos Reis

**Affiliations:** 1 Oncology, Faculdade Atenas Passos, Passos, BRA; 2 Biomedical Sciences, Hospital das Clínicas da Faculdade de Medicina da Universidade de São Paulo e Hospital Moriah, São Paulo, BRA; 3 Anesthesiology, Hospital Moriah - SP, São Paulo, BRA; 4 Pediatrics, Hospital Moriah - SP, São Paulo, BRA; 5 Genetics, Hospital das Clínicas da Faculdade de Medicina da Universidade de São Paulo e Hospital Moriah, São Paulo, BRA

**Keywords:** body mass index, obesity, prostate cancer, radical prostatectomy, surgical outcomes

## Abstract

Introduction

Elevated BMI, including overweight and obesity, is increasingly recognized as a potential risk factor for the development and progression of prostate cancer (PCa). However, its influence on perioperative, pathological, and postoperative outcomes following radical prostatectomy remains a subject of debate. This study sought to evaluate the association between elevated BMI and the clinical, surgical, pathological, and postoperative outcomes in patients undergoing robot-assisted radical prostatectomy.

Methods

This retrospective observational study included patients with histologically confirmed PCa who underwent robot-assisted radical prostatectomy at a private tertiary care hospital in Brazil between September 2017 and August 2025. Patients were stratified into two groups according to BMI: normal weight (BMI < 25 kg/m²) and elevated BMI (BMI ≥ 25 kg/m²), the latter comprising both overweight and obese individuals based on the World Health Organization (WHO) criteria. Demographic, clinical, intraoperative, pathological, laboratory, and postoperative variables were analyzed and compared between the groups. Missing data were addressed through an available-case analysis. Statistical analyses were performed using appropriate parametric and non-parametric tests, with a p-value < 0.05 considered statistically significant.

Results

A total of 1,366 patients were included in the analysis. Elevated BMI was significantly associated with a higher prevalence of systemic arterial hypertension (SAH) and diabetes mellitus (both p < 0.001). No significant associations were identified between BMI category and pathological characteristics, including pathological stage, extraprostatic extension, lymph node involvement, International Society of Urological Pathology (ISUP) grade group, seminal vesicle invasion, surgical margin status, tumor volume, or postoperative prostate-specific antigen (PSA) levels. Likewise, no significant differences were observed in intraoperative variables or in most postoperative outcomes. However, patients with elevated BMI were more likely to experience absence of spontaneous postoperative diuresis (p = 0.017). The incidence of postoperative complications did not differ significantly between the groups.

Conclusions

Elevated BMI was associated with a higher prevalence of metabolic comorbidities but was not linked to significant differences in pathological, intraoperative, or most postoperative outcomes following robot-assisted radical prostatectomy. These findings indicate that excess body weight alone may not negatively affect short-term surgical or pathological outcomes in this population. Further prospective multicenter studies are warranted to elucidate the impact of elevated BMI on PCa prognosis.

## Introduction

Prostate cancer (PCa) is the second most frequently diagnosed malignancy and remains one of the leading causes of cancer-related mortality among men worldwide. According to global estimates, approximately 1.4 million new cases and more than 375,000 deaths were reported in 2020, underscoring the substantial public health burden of this disease [1]. Moreover, projections indicate that prostate cancer mortality will continue to increase over the coming decades, particularly in low- and middle-income countries, where limited access to early detection and screening programs is associated with delayed diagnosis and poorer clinical outcomes [2].

Several factors have been associated with the incidence and progression of PCa, including advanced age, ethnicity, family history, and genetic predisposition [3]. Among the potentially modifiable risk factors, excess body weight, encompassing both overweight and obesity, has gained increasing attention because of its growing worldwide prevalence and its potential influence on cancer development and progression. Elevated BMI is associated with endocrine and metabolic disturbances, including insulin resistance, chronic low-grade inflammation, dysregulated adipokine secretion, altered lipid metabolism, and increased oxidative stress. Together, these mechanisms may promote carcinogenesis, tumor progression, and resistance to treatment. In addition, excess adiposity may increase the technical complexity of radical prostatectomy, interfere with surgical exposure, prolong operative time, and reduce serum prostate-specific antigen (PSA) concentrations through plasma hemodilution, potentially delaying diagnosis [4].

Although the relationship between excess body weight and PCa has been extensively investigated, its impact on pathological characteristics and perioperative outcomes remains controversial. Several studies have reported an association between elevated BMI and adverse oncological outcomes, including biochemical recurrence, aggressive pathological features, and PCa-specific mortality, whereas others have failed to demonstrate consistent associations [5]. These discrepancies may be explained by differences in study design, patient populations, BMI classifications, and outcome definitions, highlighting the need for additional evidence from contemporary surgical cohorts.

Beyond its potential biological effects on tumor behavior, excess body weight may also affect perioperative management and postoperative recovery following radical prostatectomy. Increased visceral adiposity may impair surgical visualization, increase technical difficulty, and contribute to perioperative morbidity. However, the available evidence remains inconsistent, and the independent impact of elevated BMI on surgical and pathological outcomes has not been fully established. Therefore, this retrospective observational study aimed to evaluate the association between elevated BMI (≥25 kg/m²) and clinical characteristics, perioperative variables, pathological findings, and postoperative outcomes in patients undergoing robot-assisted radical prostatectomy for PCa. By comparing patients with normal BMI and those with elevated BMI, this study sought to determine whether excess body weight is associated with differences in surgical performance, pathological characteristics, and early postoperative outcomes within a large contemporary cohort.

## Materials and methods

Study design

This retrospective observational cohort study was conducted at Hospital Moriah, São Paulo, Brazil. It included consecutive patients who underwent robot-assisted radical prostatectomy for histologically confirmed PCa between September 2017 and August 2023. The study was designed to evaluate the association between elevated BMI and clinical, pathological, perioperative, and postoperative outcomes. Clinical, pathological, laboratory, and follow-up data were retrospectively extracted from the institutional electronic medical records through August 2025. Thus, although data collection continued until 2025, only patients who underwent surgery during the predefined inclusion period (September 2017 to August 2023) were eligible for the study.

Study population

Eligible participants were men aged 18 years or older with histologically confirmed PCa who underwent robot-assisted radical prostatectomy as their primary treatment. Patients were included if preoperative height and weight data were available for BMI calculation and if complete clinical, pathological, surgical, and postoperative information was available for the proposed analyses. Medical records were excluded if they contained insufficient anthropometric data, duplicated entries, or did not confirm the surgical procedure.

Data collection and variables analyzed

Data were extracted through a systematic review of electronic medical records using a standardized data collection form. Demographic, clinical, anthropometric, perioperative, pathological, laboratory, and postoperative variables were collected. BMI was calculated as weight in kilograms divided by the square of height in meters (kg/m²) using preoperative measurements recorded in the electronic medical records. For analytical purposes, patients were classified into two groups according to BMI: normal weight (BMI < 25 kg/m²) and elevated BMI (BMI ≥ 25 kg/m²). The elevated BMI group included both overweight and obese patients according to the World Health Organization (WHO) classification.

The variables analyzed included age, American Society of Anesthesiologists (ASA) physical status classification, systemic arterial hypertension (SAH), diabetes mellitus, smoking status, alcohol consumption, sedentary lifestyle, pathological findings, PSA levels, intraoperative characteristics, postoperative outcomes, and postoperative complications. Missing data were handled using an available-case analysis. Each variable was analyzed using all available observations, and patients with missing information for a specific variable were excluded only from the corresponding analysis while remaining eligible for all other analyses. Consequently, the number of observations varied across variables according to data availability.

Database

Data extracted from electronic medical records and laboratory reports were entered into a dedicated Microsoft Excel database by trained investigators. The dataset underwent standardized quality control procedures, including verification of data consistency, coding standardization, and identification of duplicate or inconsistent records before statistical analysis. Continuous and categorical variables were organized according to their predefined classifications to ensure consistency throughout the analyses. As this was a retrospective study based on routinely collected clinical data, the completeness of individual variables varied across patients. Consequently, analyses were performed using an available-case approach, and the number of observations included in each analysis is reported in the corresponding tables. The study database was stored in a secure institutional environment with access restricted to the research team. All patient identifiers were removed or replaced by coded identifiers before analysis to ensure confidentiality and compliance with institutional ethical requirements.

Ethical aspects

The study was approved by the Research Ethics Committee of Hospital Moriah (approval number: 7.183.187) and was conducted in accordance with the ethical principles of the Declaration of Helsinki. The analysis was performed under an Institutional Review Board-approved umbrella research protocol designed to investigate prognostic factors in patients with PCa using the institutional database. The approved protocol comprises several predefined retrospective subprojects derived from the same patient cohort. The present analysis specifically evaluated the association between elevated BMI and clinical, pathological, perioperative, and postoperative outcomes following robot-assisted radical prostatectomy. The study included patients who underwent surgery between September 2017 and August 2023. Clinical, pathological, laboratory, and follow-up data were retrospectively collected from the institutional electronic medical records through August 2025. Accordingly, no patients undergoing surgery after August 2023 were included in the present analysis. Because of the retrospective nature of the study and the use of anonymized data, patient confidentiality was maintained throughout the study.

Statistical analysis

Initially, descriptive statistics were performed. Continuous variables are presented as mean ± standard deviation (SD) or median (interquartile range(IQR)), according to data distribution. Categorical variables are presented as absolute frequencies and percentages. Comparisons between categorical variables were performed using Pearson's chi-square test or Fisher's exact test when expected cell counts were < 5. Continuous variables were compared using Student's t-test or the Mann-Whitney U test according to data distribution. Homogeneity of variances was assessed using Levene's test. All analyses were performed using SPSS Statistics version 15.0 (IBM Corp., Armonk, NY). Statistical significance was set at p < 0.05.

## Results

Table 1 summarizes the demographic and clinical characteristics of the study population according to BMI category. A significantly higher prevalence of SAH (p < 0.001) and diabetes mellitus (p < 0.001) was observed among patients with elevated BMI (≥ 25 kg/m²) compared with those with normal BMI (< 25 kg/m²). No statistically significant differences were found between the groups regarding age (p = 0.579), ASA physical status classification (p = 0.097), smoking status (p = 0.810), alcohol consumption (p = 0.801), or sedentary lifestyle (p = 0.087), indicating a comparable distribution of these baseline characteristics between the study groups.

**Table 1:** Comparison of clinical and demographic characteristics according to BMI category The table presents the clinical and demographic characteristics of patients according to BMI category (BMI < 25 kg/m² and BMI ≥ 25 kg/m²). Continuous variables are presented as mean ± SD, and categorical variables are presented as absolute frequencies and percentages (n (%)). Comparisons between groups were performed using Student's t-test (^**^) for continuous variables and Pearson's chi-square test (^*^) for categorical variables. The "Statistic" column reports the corresponding test statistic (χ² or t), including df when applicable. A two-sided p-value < 0.05 was considered statistically significant. Total study population: 1,366 BMI: body mass index; SD: standard deviation; df: degrees of freedom; ASA: American Society of Anesthesiologists; SAH: systemic arterial hypertension

Variable	BMI category			
	BMI < 25 kg/m²	BMI ≥ 25 kg/m²	Statistic	P-value
Age, years, mean ± SD	69.56 ± 7.64	68 ± 7.53	t = 3.36 (df = 1272)	0.579^**^
ASA status, n (%)				
ASA I patients	32 (24.1%)	101 (75.9%)	χ² = 4.67 (df = 2)	0.097^*^
ASA II patients	321 (30.9%)	717 (69.1%)		
ASA III patients	15 (22.1%)	53 (77.9%)		
SAH, n (%)				
Non-hypertensive patients	201 (37.4)	336 (62.6)	χ² = 27.19 (df = 1)	< 0.001^*^
Hypertensive patients	172 (23.9)	549 (76.1)		
Diabetes, n (%)				
Non-diabetic patients	298 (32.2)	627 (67.8)	χ² = 12.56 (df = 1)	< 0.001^*^
Diabetic patients	68 (21.7)	246 (78.3)		
Smoking, n (%)				
Non-smoking patients	288 (30.3)	661 (69.7)	χ² = 0.06 (df = 1)	0.81^*^
Smoking patients	25 (31.6)	54 (68.4)		
Alcoholics, n (%)				
Non-alcoholic patients	248 (30.0%)	580 (70.0%)	χ² = 0.06 (df = 1)	0.801^*^
Alcoholic patients	73 (30.80%)	164 (69.2%)		
Sedentary, n (%)				
Non-sedentary patients	96 (34.2%)	185 (65.8%)	χ² = 2.93 (df = 1)	0.087^*^
Sedentary patients	225 (28.7%)	559 (71.3%)		

Table 2 summarizes the pathological and laboratory characteristics according to BMI category. No statistically significant associations were observed between BMI category and any of the pathological or laboratory variables analyzed, including extraprostatic extension, pathological stage, lymph node involvement, seminal vesicle invasion, tumor volume, International Society of Urological Pathology (ISUP) grade, surgical margin status, preoperative PSA, or postoperative PSA levels (all p > 0.05). Although patients with BMI ≥ 25 kg/m² showed numerically higher frequencies of positive surgical margins (p = 0.057) and higher postoperative PSA levels (p = 0.069), these differences did not reach statistical significance and should therefore be interpreted with caution.

**Table 2:** Comparison of pathological and laboratory characteristics according to BMI category Table 2 summarizes the pathological and laboratory characteristics according to BMI category. No statistically significant differences were observed between patients with BMI < 25 kg/m² and those with BMI ≥ 25 kg/m² regarding extraprostatic extension, pathological stage, lymph node involvement, seminal vesicle invasion, tumor volume, ISUP grade, surgical margin status, preoperative PSA, or postoperative PSA levels (all p > 0.05). Patients with BMI ≥ 25 kg/m² presented numerically higher frequencies of positive surgical margins (p = 0.057) and higher postoperative PSA levels (p = 0.069); however, these differences were not statistically significant. Continuous variables are presented as mean ± SD, and categorical variables as absolute frequencies and percentages (n (%)). Comparisons between groups were performed using Pearson's chi-square test (^*^), Student's t-test (^**^), or the Mann–Whitney U test (^***^), as appropriate. The "Statistic" column reports the corresponding test statistic (χ², t, or U), including df when applicable. A two-sided p-value < 0.05 was considered statistically significant. Total study population = 1,366 BMI: body mass index; SD: standard deviation; df: degrees of freedom; ISUP: International Society of Urological Pathology; PSA: prostate-specific antigen

Variable	BMI category				
	BMI < 25 kg/m²	BMI ≥ 25 kg/m²	Statistic		P-value
Extraprostatic invasion, n (%)					
Patients without invasion	75 (30.4%)	172 (69.6%)		χ² = 0.00 (df = 1)	0.962^*^
Patients with invasion	70 (30.6%)	159 (69.4%)			
Tumor staging, n (%)					
pT2 patients	72 (30.3%)	166 (69.7%)		χ² < 0.01 (df = 1)	0.985^*^
pT3 patients	70 (30.2%)	162 (60.8%)			
Lymph node, n (%)					
Patient without affected lymph node	134 (30.0%)	313 (70.0%)		χ² = 0.24 (df = 1)	0.625^*^
Patient with affected lymph node	8 (34.8%)	15 (65.2%)			
Surgical margin, n (%)					
Patients with free margin	128 (32.3%)	268 (67.7%)		χ² = 3.63 (df = 1)	0.057^*^
Patients with compromised margins	17 (21.5%)	62 (78.5)			
Seminal vesicle, n (%)					
Patients without seminal vesicle invasion	124 (29.3%)	299 (70.7%)		χ² = 2.36 (df = 1)	0.124^*^
Patients with seminal vesicle invasion	21 (39.6%)	32 (60.4%)			
Tumor volume, cm^3^, mean ± SD	5.55 ± 5.10	6.31 ± 6.13		U = 21,660	0.412^***^
ISUP, mean ± SD	3.25 ± 1.15	3.19 ± 1.05		U = 23,167	0.663^***^
PSA, ng/mL, mean ± SD	4.62 ± 2.16	6.27 ± 4.62		t = -0.67 (df = 13)	0.488^**^
Postoperative PSA, ng/mL, mean ± SD	0.01 ± 0.00	0.08 ± 0.15		t = -0.94 (df = 10)	0.069^**^

Table 3 summarizes the intraoperative characteristics according to BMI category. Patients with BMI ≥ 25 kg/m² had numerically longer mean operative time (p = 0.481), anesthesia time (p = 0.207), and operating room stay (p = 0.189) than patients with BMI < 25 kg/m²; however, none of these differences reached statistical significance. Likewise, no statistically significant differences were observed between BMI categories regarding the need for mechanical ventilation (p = 0.212) or intraoperative blood transfusion (p = 0.885).

**Table 3:** Comparison of intraoperative characteristics according to BMI category The table presents intraoperative characteristics according to BMI category (BMI < 25 kg/m² and BMI ≥ 25 kg/m²). Continuous variables are presented as mean ± SD, and categorical variables as absolute frequencies and percentages (n (%)). Comparisons between groups were performed using Student's t-test (^**^) for continuous variables and Pearson's chi-square test (^*^) for categorical variables. The "Statistic" column reports the corresponding test statistic (χ² or t), including df when applicable. A two-sided p-value < 0.05 was considered statistically significant. Total study population: 1,366 BMI: body mass index; SD: standard deviation; df: degrees of freedom

Variable	BMI category			
	BMI < 25 kg/m²	BMI ≥ 25 kg/m²	Statistic	P-value
Surgery time, minutes, mean ± SD	179.57 ± 85.02	183.61 ± 51.21	t = -1.04 (df = 1270)	0.481^**^
Anesthesia time, minutes, mean ± SD	216.74 ± 52.72	225.59 ± 53.77	t = -2.69 (df = 1271)	0.207^**^
Operating room stay, minutes, mean ± SD	244.90 ± 53.89	252.39 ± 55.85	t = -2.21 (df = 1271)	0.189^**^
Mechanical ventilation, n (%)				
Patients without ventilation	16 (36.4)	28 (63.6)	χ² = 1.56 (df = 1)	0.212^*^
Patients with ventilation	229 (27.7)	598 (72.3)		
Blood infusion, n (%)				
Patients who did not receive infusion	363 (29.5%)	867 (70.5%)	χ² = 0.021 (df = 1)	0.885^*^
Patients who received infusion	1 (33.3%)	2 (66.7%)		

Table 4 summarizes the postoperative outcomes according to BMI category. A statistically significant difference was observed between BMI categories regarding the absence of spontaneous postoperative diuresis (p = 0.017), with a higher frequency among patients with BMI ≥ 25 kg/m². No statistically significant differences were identified for the remaining postoperative outcomes, including ICU admission (p = 0.210), hospital readmission (p = 0.684), length of hospital stay (p = 0.909), post-anesthesia care unit (PACU) stay after discharge (p = 0.121), or total PACU stay until discharge (p = 0.111). Data regarding urinary catheterization time were not analyzed because of incomplete information in the electronic medical records.

**Table 4:** Comparison of postoperative outcomes according to BMI category The table presents postoperative outcomes according to BMI category (BMI < 25 kg/m² and BMI ≥ 25 kg/m²). Continuous variables are presented as mean ± SD, and categorical variables as absolute frequencies and percentages (n (%)). Comparisons between groups were performed using Pearson's chi-square test (^*^), Student's t-test (^**^), or the Mann–Whitney U test (^***^), as appropriate. The "Statistic" column reports the corresponding test statistic (χ², t, or U), including df when applicable. Data regarding urinary catheterization time were excluded from the analysis because of incomplete electronic medical records. A two-sided p-value < 0.05 was considered statistically significant. Total study population = 1,366 BMI: body mass index; ICU: intensive care unit; SD: standard deviation; df: degrees of freedom; PACU: post-anesthesia care unit

Variable	BMI category			
	BMI < 25 kg/m²	BMI ≥ 25 kg/m²	Statistic	P-value
ICU stay, n (%)				
Patients who were not admitted to the ICU	373 (29.7%)	885 (70.3%)	χ² = 1.57 (df = 1)	0.21^*^
Patients who were admitted to the ICU	2 (14.3%)	12 (85.7%)		
Spontaneous diuresis, n (%)				
Patients who had spontaneous diuresis	15 (44.1%)	19 (55.9%)	χ² = 5.73 (df = 1)	0.017^*^
Patients who did not have spontaneous diuresis	50 (24.4%)	155 (75.6%)		
Hospital readmission, n (%)				
Patients who did not require readmission	128 (31.8%)	275 (68.2%)	χ² = 0.17 (df = 1)	0.684^*^
Patients who required readmission	2 (25%)	6 (75%)		
Length of stay, days, mean ± SD	2.33 ± 0.54	2.32 ± 0.57	t = 0.20 (df = 1272)	0.909^**^
Stay in PACU after discharge, minutes, mean ± SD	9.47 ± 14.51	12.86 ± 30.81	U = 118,744	0.121^***^
Stay in PACU until discharge, minutes, mean ± SD	57.48 ± 34.05	59.86 ± 36.1	t = -1.06 (df = 1191)	0.111^**^

Table 5 summarizes the postoperative complications according to BMI category. Postoperative complications, including fistula, infection, venous thromboembolism (VTE), bleeding, and erectile dysfunction, were infrequent in both groups. No statistically significant differences were observed between patients with BMI < 25 kg/m² and those with BMI ≥ 25 kg/m² for any of the postoperative complications analyzed (all p > 0.05).

**Table 5:** Comparison of postoperative complications according to BMI category The table presents postoperative complications according to BMI category (BMI < 25 kg/m² and BMI ≥ 25 kg/m²). Data are presented as absolute frequencies and percentages (n (%)). Comparisons between groups were performed using Pearson's chi-square test or Fisher's exact test, as appropriate. The "Statistic" column reports the corresponding test statistic, including df when applicable. A two-sided p-value < 0.05 was considered statistically significant. Total study population = 1,366 BMI: body mass index; df: degrees of freedom; PTE: pulmonary thromboembolism; DVT: deep vein thrombosis

Variable	BMI category			
	BMI < 25 kg/m²	BMI ≥ 25 kg/m²	Statistic	P-value
Fistula, n (%)				
Patients who did not have a fistula	375 (29.6%)	894 (70.4%)	χ² = 0.42 (df = 1)	0.517
Patients who had a fistula	0 (0%)	1 (100%)		
Infection, n (%)				
Patients without infection	371 (29.6%)	883 (70.4%)	χ² = 1.26 (df = 1)	0.262
Patients who had an infection	0 (0%)	3 (100%)		
PTE/DVT, n (%)				
Patients who did not have PTE/DVT	376 (29.6%)	896 (70.4%)	χ² = 0.42 (df = 1)	0.517
Patients who had PTE/DVT	0 (0%)	1 (100%)		
Bleeding, n (%)				
Patients who did not bleed	341 (29.4%)	819 (70.6%)	χ² = 0.02 (df = 1)	0.878
Patients who experienced bleeding	3 (27.3%)	8 (72.7%)		
Sexual impotence, n (%)				
Patients without sexual impotence	318 (29.6%)	756 (70.4%)	χ² = 0.02 (df = 1)	0.888
Patients with sexual impotence	1 (33.3%)	2 (66.7%)		

## Discussion

This retrospective study evaluated the association between elevated BMI (≥ 25 kg/m²) and clinical, pathological, and perioperative outcomes in a cohort of 1,366 patients undergoing robot-assisted radical prostatectomy. Overall, elevated BMI was not associated with significant differences in pathological characteristics, intraoperative variables, or most postoperative outcomes. However, patients with elevated BMI had a significantly higher prevalence of systemic arterial hypertension and diabetes mellitus, reinforcing the well-established relationship between excess body weight and cardiometabolic comorbidities. These findings also highlight the heterogeneity of the available literature regarding the impact of increased BMI on PCa outcomes [6,7].

The higher prevalence of hypertension and diabetes observed among patients with elevated BMI is consistent with previous reports demonstrating the close association between excess body weight and metabolic disorders [8]. Obesity and overweight are characterized by chronic low-grade inflammation, insulin resistance, endothelial dysfunction, and metabolic alterations that contribute not only to cardiovascular disease but may also influence cancer biology [9]. Consequently, patients with elevated BMI frequently present with a greater burden of comorbidities at the time of PCa diagnosis, which may affect perioperative management and postoperative recovery.

From a biological perspective, adipose tissue functions as an active endocrine organ capable of secreting adipokines, inflammatory cytokines, and growth factors that contribute to a pro-inflammatory and pro-tumorigenic microenvironment. Chronic inflammation, oxidative stress, and activation of signaling pathways such as PI3K/AKT/mTOR have been implicated in tumor initiation, progression, and therapeutic resistance in PCa [10,11]. Furthermore, previous studies have suggested that lower BMI may be associated with improved cancer-specific survival, supporting the hypothesis that excess adiposity may influence long-term oncological outcomes [12].

Despite these biological mechanisms, no statistically significant differences were observed between BMI categories regarding pathological stage, extraprostatic extension, lymph node involvement, ISUP grade, seminal vesicle invasion, tumor volume, or surgical margin status. These findings differ from some previous studies and meta-analyses reporting an association between obesity and more aggressive pathological features or increased PCa mortality [6]. Several factors may explain these discrepancies. First, the present study included only patients who underwent robot-assisted radical prostatectomy, resulting in a relatively homogeneous population with localized disease and favorable surgical candidacy. Consequently, the prevalence of advanced disease was relatively low, which may have reduced the ability to detect differences in pathological outcomes. In addition, BMI is an indirect measure of adiposity and does not distinguish lean body mass from fat mass or quantify visceral adipose tissue, which has been more strongly associated with adverse metabolic and oncological outcomes [9]. Therefore, BMI alone may not adequately reflect the biological mechanisms linking excess adiposity to PCa aggressiveness.

Another important consideration is the potential influence of elevated BMI on serum PSA concentrations. Increased plasma volume in overweight and obese individuals may lead to PSA hemodilution, resulting in lower circulating PSA levels and potentially delaying PCa detection [13]. This phenomenon may influence preoperative risk stratification and partially explain the absence of significant differences in pathological characteristics between BMI categories observed in the present study. Consequently, the results should be interpreted considering the inherent limitations of BMI as a surrogate marker of adiposity and the differences in study design, patient selection, and clinical outcomes evaluated across the literature.

Although patients with BMI ≥ 25 kg/m² presented numerically higher frequencies of positive surgical margins and higher postoperative PSA levels, these differences did not reach statistical significance. Therefore, these findings should not be interpreted as evidence of an association between elevated BMI and adverse oncological outcomes in the present cohort. Nevertheless, positive surgical margins and detectable postoperative PSA remain clinically relevant prognostic factors because of their established association with biochemical recurrence after radical prostatectomy [14]. Future studies with larger sample sizes and longer follow-up may help clarify whether these numerical differences represent clinically meaningful findings.

The absence of statistically significant differences in surgical margin status may also reflect the widespread use of robot-assisted radical prostatectomy in this cohort. Robotic surgery provides enhanced visualization, greater instrument dexterity, and improved precision during dissection, potentially minimizing the technical challenges traditionally associated with excess body weight. Consequently, the impact of elevated BMI on surgical performance may be less pronounced than previously reported in studies that included open surgical approaches.

A significant difference was observed regarding the absence of spontaneous postoperative diuresis, which was more frequent among patients with BMI ≥ 25 kg/m². Although the underlying mechanism cannot be established from the present study, this finding may reflect the physiological changes commonly associated with excess body weight, including endothelial dysfunction, chronic inflammation, altered renal perfusion, and activation of the renin-angiotensin-aldosterone system [7,15]. These mechanisms have been associated with impaired renal adaptation to surgical stress and may contribute to transient postoperative urinary dysfunction. However, given the observational nature of this study, this finding should be interpreted cautiously and requires confirmation in prospective investigations.

This study has several limitations. First, the retrospective design makes the study susceptible to selection bias, information bias, and residual confounding. Second, all patients were treated at a single tertiary referral center, which may limit the generalizability of the findings. Third, BMI was used as the sole anthropometric measure of adiposity. Although BMI is widely used in clinical practice, it does not distinguish lean body mass from fat mass or provide information regarding visceral fat distribution, which may be more strongly associated with PCa aggressiveness. In addition, some variables contained missing data because information was extracted from routinely collected electronic medical records. To minimize potential bias, an available-case analysis was performed, and each variable was analyzed using all available observations. Finally, the study evaluated primarily short-term perioperative and pathological outcomes, without long-term follow-up for biochemical recurrence, cancer-specific survival, or overall survival.

Despite these limitations, the present study has important strengths. It included a large contemporary cohort of patients undergoing robot-assisted radical prostatectomy, with standardized surgical management and comprehensive evaluation of clinical, pathological, intraoperative, and postoperative variables. These characteristics provide valuable real-world evidence regarding the influence of elevated BMI on perioperative outcomes in patients with localized PCa.

Overall, the findings suggest that elevated BMI is associated with a greater burden of cardiometabolic comorbidities but does not appear to substantially influence pathological findings, perioperative variables, or most short-term postoperative outcomes following robot-assisted radical prostatectomy. Further prospective multicenter studies incorporating more accurate measures of body composition and long-term oncological follow-up are warranted to better define the relationship between excess adiposity and PCa outcomes.

## Conclusions

The findings of this study indicate that elevated BMI (≥ 25 kg/m²) was not associated with significant differences in pathological, surgical, or most postoperative outcomes among patients undergoing robot-assisted radical prostatectomy for PCa. Patients with elevated BMI had a higher prevalence of metabolic comorbidities, particularly systemic arterial hypertension and diabetes mellitus, and were more likely to experience absence of spontaneous postoperative diuresis. Overall, these findings suggest that elevated BMI alone may not adversely affect short-term perioperative or pathological outcomes in this population. Further prospective multicenter studies incorporating more comprehensive measures of body composition and long-term oncological follow-up are warranted to better clarify the relationship between excess body weight and PCa outcomes.
